# Online Personas: Associations Between Focus on Self-Presentation and Social Comparison on Social Media and Mental Well-Being in Early Adolescence

**DOI:** 10.3389/ijph.2025.1608425

**Published:** 2025-04-30

**Authors:** Rachana Aryal, Gunnhild Johnsen Hjetland, Ellen Haug, Oddrun Samdal, Jens Christoffer Skogen

**Affiliations:** ^1^ Department of Health Promotion, Norwegian Institute of Public Health, Bergen, Norway; ^2^ Centre for Evaluation of Public Health Measures, Norwegian Institute of Public Health, Oslo, Norway; ^3^ Department of Health Promotion and Development, Faculty of Psychology, University of Bergen, Bergen, Norway; ^4^ Department of Teacher Education, NLA University College, Bergen, Norway; ^5^ Center for alcohol and drug research (KORFOR), Stavanger University Hospital, Stavanger, Norway

**Keywords:** social media use, self-presentation, social comparison, wellbeing, adolescence

## Abstract

**Background:**

The development of identity and social interactions during adolescence is increasingly intertwined with social media use. This study examines the relationship between focus on self-presentation on social media and wellbeing among Norwegian adolescents aged 13–15.

**Methods:**

Data from the 2022 Health Behaviour in School-Aged Children (HBSC) survey, encompassing 1,982 participants. Wellbeing was assessed using the WHO-5 Wellbeing Index, while self-presentation focus was measured using the Self-Presentation and Upward Social Comparison Inclination Scale (SPAUSCIS). Statistical models for estimating unadjusted and adjusted associations were employed, as well as testing for age- and gender-moderation.

**Results:**

Higher focus on self-presentation was associated with lower wellbeing (unadjusted: β −5.1, p < 0.001; fully adjusted: β −2.5, p < 0.001). The association was stronger for girls (unadjusted: β −4.6, p < 0.001) than boys (unadjusted: β −2.0, p < 0.001). Gender-moderation was significant (p = 0.012), but no age-moderation was observed (p = 0.057).

**Conclusion:**

The findings indicate a negative association between focus on self-presentation and wellbeing, with a more pronounced effect observed in girls compared to boys. The study underscores the need for public health interventions targeting the reduction of self-presentation and social comparison behaviors on social media.

## Introduction

Adolescence is a life-changing developmental stage marked by profound social, emotional, and physical changes [1]. Adolescents aspire to establish their identities, gain independence, and build social relationships [1, 2]. These developmental tasks are essential for understanding one’s role in the world and developing a sense of self [2]. However, the pervasive use of social media may have introduced new challenges to the completion of these developmental tasks [3]. Social media platforms now play a crucial role in how adolescents express their identities, handle relationships, and engage in social comparison [4]. This growing reliance on social media has raised concerns among parents, educators, and researchers about the potential impact of social media on the mental wellbeing and overall wellbeing of young people [5]. As adolescents strive to answer fundamental questions of identity and belonging, how social media intersects with these developmental tasks remains an important area of study.

Initially, research on social media and associations with mental wellbeing focused on the amount of time spent on these platforms. Early studies suggested that the excessive use of social media could be contributing to negative mental wellbeing outcomes such as anxiety and depression, although the identified associations were generally small and of questionable practical significance [6, 7], and a 2024 meta-analysis concludes that the existing evidence does not support overall harmful effects of time spent on social media [8]. More recent primary studies has shifted towards understanding the specific aspects of social media use that might be more directly related to mental wellbeing outcomes [9, 10]. This shift acknowledges that different aspects of social media use, certain motivations, experiences, behaviors, and interactions on these platforms may have varying effects [10–12]. One such area of focus has been self-presentation and social comparison on social media. Online self-presentation involves the ways individuals curate and manage their online personas to influence how others perceive them [1, 13]. This curation often intersects with upward social comparison, i.e., comparing oneself to someone who is viewed as better, which can often lead to feelings of inadequacy or low self-esteem [14].

The gender perspective is generally considered important in relation to media and motivations and potential effects [15]. And gender may be an especially pertinent factor to consider when it comes to social media, self-presentation and social comparison [16]. Social psychological processes drive the behavior of self-presentation, such as the process of creating and maintaining one’s online image [17]. Immediate feedback in the form of likes and comments can make individuals more conscious of their online image, while the ability to reach a broad audience can amplify the pressure to present an idealized version of oneself [12, 18]. Similarly, social media platforms can greatly amplify social comparisons and their adverse emotional effects due to their emphasis on presenting idealized lives and bodies, highly visual content, and interaction with a global audience [10, 19]. These social media-related mechanisms are likely intertwined in with broader media factors such as stereotypic gender representations, highlighting for instance physical appearance when it comes to women [20].

Previous studies have highlighted the complex relationship between self-presentation and upward social comparison on social media and mental wellbeing outcomes. This complexity results from people’s diverse approaches to social comparison and self-presentation, which can have both beneficial and adverse effects depending on the situation. For example, promoting authentic self-presentation—where individuals represent their true selves—has been associated with higher levels of psychological wellbeing and self-esteem [21]. Conversely, studies on Norwegian high school students have found that a higher focus on self-presentation, including feedback-seeking, strategic self-presentation, and upward social comparison, was associated with increased mental health problems and reduced quality of life among adolescents [6, 22]. Similarly, poor mental wellbeing outcomes such as anxiety and depression can result when self-presentation becomes more idealized or planned, including behaviors like upward social comparison and excessive feedback-seeking [23]. Another study identified specific sociodemographic and personality traits linked to a higher focus on self-presentation, suggesting that certain groups may be more susceptible to the negative effects of social media. Specifically, the study found that, overall, a higher focus on self-presentation was associated with being female, having higher levels of extraversion, lower emotional stability, more frequent alcohol consumption, and having tried tobacco, highlighting both sociodemographic and lifestyle factors [5]. Understanding these factors is essential for developing targeted intervention strategies to mitigate the adverse effects of self-presentation behaviors [5]. Likewise, findings from another study also show that as children transition into adolescence, girls who engage more with social media content centered around peers experience lower self-esteem, highlighting their particular vulnerability during this developmental period [24]. Additionally, a study among adolescents aged 11–18 years explored how social media content created by peers may impact adolescents’ self-imagery and self-esteem, further emphasizing the need to understand these multi-faceted dynamics in the context of mental wellbeing [11].

Despite these insights in relation to online self-presentation and social comparison, gaps remain in the existing literature. Most studies have focused on older adolescents, leaving a need to understand how these behaviors play out among younger adolescents. The present study aims to fill this gap by investigating the association between focus on self-presentation and mental wellbeing among adolescents aged 13–15 years. Using data from the nationally representative Norwegian part of the “Health Behaviour in School-Aged Children Study” (HBSC), this research will be the first to explore these associations in this younger adolescent age group. Additionally, the study will examine potential age- and gender-moderation, as well as the role of sociodemographic factors and problematic social media use as potential confounders on the estimated association. Results from this study will contribute to a greater understanding of how online self-presentation may impact the mental wellbeing of younger adolescents, potentially informing future interventions and policies aimed at promoting healthier social media use among adolescents.

## Methods

### Sample

The data employed in the present study is derived from the Norwegian part of the “Health Behavior in the School-Aged children Study” (HBSC) of 2022 [25]. The survey collected data from 11-, 13-, 15-, and 16-years-old. The HBSC study focuses on health behaviors, health perceptions, wellbeing, and contextual factors among children and adolescents. The data collection is conducted using self-administered questionnaires during school hours, under the supervision of teachers following guidelines from the research team. By employing a systematic cluster sampling method, the survey aims to establish a nationally representative sample of school-aged children [26]. The study and the use of passive parental was approved by the Norwegian South-Eastern Regional Ethical Committee (REK#346737). The participants were informed that participation was voluntary and that they could withdraw their consent at any point. For the purposes of the present study only those aged 13 and 15 years were included making a sample size of N = 1982, as they were the only two age groups receiving items covering focus on self-presentation and social comparison on social media.

### Measures

#### Socidemographics

Age-category was determined by the participants responses to which school grade they belonged to [8th grade (13 years) or 10th grade (15 years)], and gender was based on self-report, choosing between “boy” and “girl”. Subjective socioeconomic status (S-SES) was measured using an adolescent-specific version of the MacArthur Scale of Subjective Social Status. The question consists of a ladder with ten steps based on the how the Norwegian society is set up. The participants were asked to mark the rung that most aligned with their assessment of where their family belong from “worst off” to “best off” (range 0–10) [27]. S-SES was used a continuous variable.

#### Mental Wellbeing: WHO-5 Wellbeing Index

The WHO-5 Wellbeing Index was used to assess mental wellbeing. The index consists of five statements regarding mental wellbeing over the last 2 weeks, each on a six-level ordinal scale (range 0–5). The five individual item scores were summed and the total was then multiplied by 4 to obtain a final score with a potential range from 0 to 100 (www.who-5.org). A higher score indicates a higher level of mental wellbeing. For the present study, the score was employed as a continuous variable, although recommended cut-points for a categorization exist [28]. Cronbach’s *α* was 0.84 in the study sample, indicating a high internal reliability.

#### Focus on Self-Presentation: Self-Presentation and Upward Social Comparison Inclination Scale (SPAUSCIS)

**FIGURE 1:**
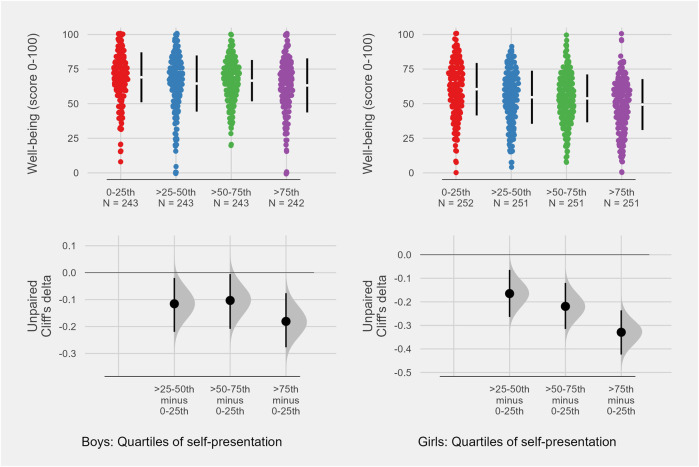
Estimation plot. Association between quartiles of self-presentation on social media and well-being. Lower panel indicates effect sizes in Cliff‘s Delta. N =1982. Data from Health Behavior in the School-Aged children Study (HBSC; Norway. 2022).

Focus on self-presentation and upward social comparison on social media (referred to as “focus on self-presentation” in the remainder) was assessed using SPAUSCIS. SPAUSCIS consists of 7 items on a five-level ordinal scale (range 1–5). The psychometric properties have been investigated in two independent samples of adolescents, and both indicated a unidimensional construct with high reliability [5, 6]. In the present study, Cronbach’s *α* was 0.90, indicating a high internal reliability. SPAUSCIS was also unidimensional in the present sample based on results from exploratory factor analysis and Mokken scale analysis (see Supplementary Appendix SA for results from factor analysis and Mokken scale analysis). For the purposes of the present study, the average score of the summed seven individual items was computed. In the main analyses, SPAUSCIS was used as both a continuous measure (Table 2) and as a categorical variable divided into quartiles (Figure 1). A higher score on SPAUSCIS indicates a higher level of focus on self-presentation (range 1–5).

#### Problematic Social Media Use (PSMU): The Social Media Disorder Scale

The Social Media Disorder Scale was used to gauge potential problematic use of social media [29]. The scale consists of nine questions covering the commonly used criteria for PSMU. The response categories are either “Yes” or “No,” and to be classified as a “problematic user” an affirmative response to six or more questions was required. In the present study, we constructed a binary variable that differentiated between “problematic user” and “non-problematic user” as per recommendations [29]. Cronbach’s *α* was 0.78 in the study sample, indicating adequate internal reliability.

### Statistical Analyses

First, the descriptive statistics of the included variables across boys and girls for the two age groups combined were calculated and presented across boys and girls (Table 1). For age, S-SES, self-presentation on social media and mental wellbeing, the median and interquartile range was computed, and for problematic social media use the frequency proportion was computed. For the main analysis investigating the association between self-presentation as continuous measure and mental wellbeing, multiple multilevel mixed effects linear regression models were employed. All these models accounted for clustering by estimating random intercepts for school and class, and the Kenward-Roger approximation was used for degrees of freedom [30]. Initial overall association between focus on self-presentation and mental wellbeing were computed across hierarchical models (data not shown): an unadjusted model; a model adjusted for age; a model adjusted for age and gender, a model adjusted for age, gender and subjective socioeconomic status; a model adjusted for age, gender, subjective socioeconomic status and problematic social media use (fully adjusted model). As a sensitivity test, each individual item of the SPAUSCIS in relation to mental wellbeing for the overall sample was computed for the unadjusted and the fully adjusted model (see Supplementary Appendix SB). Potential moderation effects of age and gender on the estimated associations were examined separately using one-way interaction analyses in multilevel models. In these models, SPAUSCIS was also treated as a continuous variable, and adjustments were made for subjective socioeconomic status. Depending on the focus of the moderation effect, the models also included adjustments for either age or gender, ensuring that the analyses appropriately accounted for potential confounding factors. Likelihood ratio tests were used, comparing models with and without the interaction term. Moderation was considered present at *p <* 0.05, and stratified results were presented. Due to evidence for gender moderation (see results section), gender-stratified hierarchical models were computed next: unadjusted models; models adjusted for age; models adjusted for age and subjective socioeconomic status; models adjusted for age, subjective socioeconomic status and problematic social media use (fully adjusted model). These gender-stratified models constitute the main analyses in our study (Table 2).

**TABLE 1:** Descriptive statistics. N = 1982. Data from Health Behavior in the School-Aged children Study (HBSC; Norway. 2022).

Variable	Boys (N = 975^a^)	Girls (N = 1,007^a^)	Difference^b^	95% CI^b^ ^,^ ^c^	p-value^b^
Age	14.33 (13.75, 15.67)	14.25 (13.75, 15.75)	0.01	−0.08, 0.10	0.8
S-SES (range 0–10)	8.00 (8.00, 9.00)	8.00 (7.00, 9.00)	0.10	−0.05, 0.25	0.2
Problematic SoMe use	61 (6.4%)	111 (11%)	−4.8%	−7.4%, −2.2%	<0.001
Self-presentation on social media (range 1–5)	1.14 (1.00, 1.71)	1.71 (1.29, 2.43)	−0.44	−0.51, −0.37	<0.001
Wellbeing (score 0–100)	68 (56, 76)	56 (44, 68)	11	9.6, 13	<0.001

^a^ Median (Q1, Q3); n (%).

^b^ Welch Two Sample t-test; 2-sample test for equality of proportions with continuity correction.

^c^ CI, confidence interval.

**TABLE 2:** Association between self-presentation (SPAUSCIS; independent variable) and mental wellbeing (WHO-5 Wellbeing Index; dependent variable). Gender-stratified multilevel mixed effects linear regression. N = 1982. Data from Health Behavior in the School-Aged children Study (HBSC; Norway. 2022).

Model	Boys (N = 975)			Girls (n = 1,007)		
	Coefficient (β)	95% CI^*a*^	p-value	Coefficient (β)	95% CI^*a*^	p-value
Unadjusted	−2.0	−3.6, −0.50	0.010	−4.6	−6.0, −3.2	<0.001
Adjusted for age	−2.0	−3.5, −0.49	0.010	−4.6	−6.0, −3.2	<0.001
Adjusted for age and S-SES	−1.6	−3.1, −0.20	0.026	−4.0	−5.4, −2.6	<0.001
Fully adjusted	−1.8	−3.4, −0.22	0.027	−3.3	−4.7, −1.8	<0.001

^a^ CI, confidence interval.

S-SES: Subjective socioeconomic status. Fully adjusted: Age, S-SES and problematic social media use.

Additionally, the crude association between self-presentation and mental wellbeing was computed and presented (Figure 1) as estimation plots using bootstrap-coupled estimation (DABEST) for boys and girls separately. DABEST offers several benefits over traditional null-hypothesis significance testing by focusing on the effect size and reducing dichotomous thinking inherent in the latter [31]. In the present study, Cliff’s delta [32] with bootstrapped 95% confidence intervals was computed using *n* = 5,000 bootstrap samples. All data analyses were performed using R Studio, with the following packages: “gtsummary” [33], “dabestr” [31], “mokken” [34, 35], “psych” [36], “lme4” [37] and “lmerTest” [38].

## Results

Descriptive statistics are presented in Table 1. There was no difference in age or S-SES between boys and girls (p-values > 0.05). Boys reported a lower score for focus on self-presentation and a lower rate of problematic social media use than girls (p-values < 0.001), and a higher score on wellbeing (p-value < 0.001).

### Association Between Focus on Self-Presentation and Mental Wellbeing

There was an association between higher levels of focus on self-presentation and lower wellbeing across adjustments in the total sample [slope: *β* −5.1 (*p <* 0.001) in the unadjusted versus *β* −2.5 (*p <* 0.001) in the fully adjusted model]. Only adjustment for gender meaningfully changed the estimated association, and the strength of the association was substantially reduced [age-adjusted model *β* −5.0 (*p <* 0.001) vs. age-and-gender-adjusted model *β* −3.4 (*p <* 0.001); a 32% reduction in the estimated association].

Based on the likelihood-ratio test, no age-moderation was observed (*p* = 0.057). However, the likelihood-ratio test for gender-moderation was significant (*p* = 0.012). The main results and from the bootstrap-coupled estimation (DABEST) were therefore presented stratified by gender (Table 2; Figure 1).

For both boys and girls, an association between higher levels of self-presentation and lower levels of wellbeing was observed across adjustments, but the association was weaker for boys compared to girls (Table 2). The estimated association were mostly unaffected across adjustments for boys (i.e., *β* −2.0 in unadjusted model vs. *β* −1.8 in fully adjusted model), while the association was quite substantially weakened in the full adjusted model for girls (i.e., *β* −4.6 in unadjusted vs. *β* −3.3 in fully adjusted model).

A similar overall association was found in the bootstrap-coupled estimation using self-presentation on social media as quartiles (Figure 1). For boys, the largest effect size of *δ* −0.18 (95%CI: −0.28, −0.08) was between the lowest and the highest quartile of self-presentation, while the corresponding effect size was *δ* −0.33 (95%CI: −0.42, −0.24) for girls.

## Discussion

This study shows a clear association between heightened self-presentation on social media and decreased mental wellbeing in adolescents aged 13 and 15. The study found a significant moderation effect by gender, showing a stronger link between self-presentation and mental wellbeing in girls. Using bootstrap-coupled estimation (DABEST), a moderate effect size was observed for girls, while boys showed a smaller effect size, highlighting the gender disparity. These findings highlight the need to recognize gender-specific vulnerabilities in digital engagement and suggest that interventions should be tailored to address these unique challenges.

The results demonstrate a significant association between higher levels of focus on self-presentation and lower mental wellbeing, consistent with previous findings in older adolescents [6, 22]. Our findings also support earlier research indicating that girls may be more susceptible to the negative effects of social media due to greater involvement in self-presentation and social comparison [22, 39].

### Comparison With Existing Literature

The findings of this study align with and extend the insights from previous research on social media use and adolescent mental wellbeing. For instance, a study performed among pupils attending senior high schools’ (16–21 years) in Bergen, Norway [5] explored how self-presentation behaviors vary across different sociodemographic and personality profiles. The findings suggested that adolescents who have higher levels of extraversion, lower emotional stability, more frequent alcohol consumption, and tried tobacco, may be more prone to engage in self-presentation behaviors that could impact their mental wellbeing. Our study extends on these findings by demonstrating that gender differences play a significant role, with girls showing a stronger association between self-presentation and reduced mental wellbeing. In contrast to our findings, Hjetland et al. [22] found no interaction effect of gender on mental health problems, suggesting that the associations between self-presentation and depression, anxiety, and wellbeing were not different for boys and girls among adolescents who were 16 years or older. A study by Orben et al. [40] highlighted that adolescents experience developmental windows of sensitivity to social media, with potential impacts on mental wellbeing varying by age and gender. This could explain differences in findings regarding focus on self-presentation for younger versus older adolescents. Whereas vulnerability to gendered effects might be more acute during early adolescence when social and emotional development is more heightened, one could expect responses among older adolescents to become more uniform across gender as they develop greater emotional regulation and social understanding.

Our findings reveal distinct gender differences in self-presentation and wellbeing, with boys reporting lower levels of self-presentation and problematic social media use, alongside higher wellbeing scores compared to girls. This aligns with previous research emphasizing the influence of social constructions and stereotypes on media use motivations [15]. Additionally, the notion that girls may engage more frequently in self-presentation due to societal norms and expectations, highlights the need to explore gender-specific media choices and patterns in self-presentation [41, 42].

Another relevant study, done among 152 adolescents aged 11–18 years in the US [11], explored how self-presentation relate to self-esteem and self-concept among adolescents. The study showed that frequent use of social media, coupled with self-presentation and social comparison behavior, may influence adolescents’ self-imagery and self-esteem. Our research adds to these findings by linking focus on self-presentation to lower mental wellbeing, suggesting reliance on online validation, approval anxiety, and increased self-objectification may potentially undermine self-esteem and self-concept clarity.

Additionally, our findings can also be understood in consideration of findings from research on authenticity and wellbeing while using social media [43]. A study by Bailey et al. found that authentic self-expression may benefit wellbeing, reinforcing the idea that active ways of using social media are important for mental health [43]. Thus, authentic self-expression counters the adverse effects of idealized self-presentation through increasing life satisfaction, building social support and belonging, improving self-concept, and reducing the negative impact of social comparison. This is one avenue to improved mental wellbeing by being more true to oneself [43]. Similarly, research on self-presentation, self-concept clarity, and depressive symptoms support these observations by suggesting that a lack of authenticity in online interactions may contribute to negative mental health outcomes, such as depressive symptoms [40, 44]. Although the referenced studies focus on older populations and specific platforms, their insights on authenticity align with our findings, emphasizing the risks associated with deceptive self-presentation and the potential benefits of authenticity. However, it is important to note that our study did not directly measure authenticity, which limits our ability to draw definitive conclusions about its role in the observed outcomes.

By building on insights from previous studies, our research underscores the complex interplay between social media behaviors and mental wellbeing outcomes. It also highlights the need for targeted interventions that can help adolescents to be less preoccupied with their self-presentation online and address the unique challenges posed by self-presentation and social comparison on social media platforms. Such efforts could play a vital role in enhancing adolescent mental wellbeing in the digital age.

### Strengths and Limitations

The study’s strengths include the use of data from the nationally representative “Health Behavior in School-Aged Children Study” (HBSC) of 2022, with a systematic cluster sampling method ensuring robust and generalizable findings within the Norwegian adolescent population. The measures used in this study were carefully selected to ensure comprehensive coverage of the variables of interest. The WHO-5 Wellbeing Index, used to measure mental wellbeing, demonstrated high internal reliability within the sample. The Self-Presentation and Upward Social Comparison Inclination Scale (SPAUSCIS) also showed high reliability, capturing the nature of focus on self-presentation on social media. Problematic social media use was evaluated using the Social Media Disorder Scale, which identified participants as either problematic or non-problematic users based on established criteria [29].

However, several limitations must be acknowledged. The cross-sectional nature of the data limits the ability to make causal inferences or determine the directionality of the associations observed [22]. The reliance on self-reported data introduces the potential for response bias, affecting the accuracy of the findings. Additionally, unmeasured confounding may be a concern, as other relevant variables influencing the associations may not have been included.

### Public Health Relevance

The findings of this study hold substantial public health relevance, particularly in the context of adolescent mental wellbeing. As social media becomes an increasingly integral part of adolescents’ lives, understanding the specific behaviors in social media use that contribute to mental wellbeing outcomes is crucial. This study sheds light on how self-presentation and social comparison on social media can negatively impact mental wellbeing, especially among younger adolescents. By focusing on these specific aspects rather than general usage metrics, such as time spent online, our study provides actionable insights for public health interventions and policymaking.

The results suggest that interventions aimed at promoting healthier social media habits should prioritize and include components addressing self-presentation and social comparison behaviors. This may be particularly important for girls, who appear to be more vulnerable to the potential negative effects associated with these behaviors. For younger adolescents, age-specific educational programs may focus on building self-esteem and reducing upward social comparisons, while older adolescents might benefit from strategies to manage impression management and self-presentation pressure [40]. Public health strategies could include educational programs that encourage authentic self-expression, less reliance on social media for self-presentation and resilience against social pressures. Such programs could be integrated into school curricula to reach adolescents effectively.

According to Andersen et al., incorporating social media and digital literacy into educational programs may be one way forward and crucial for fostering healthier social media habits among adolescents [45]. Findings from the same study also indicated that school-based programs may increase awareness and reflection on social media use, though further research is needed to assess actual effect, especially long-term effects [45]. By enhancing critical thinking and awareness about both personal and others’ social media use, these programs help young people navigate digital complexities. They teach adolescents to recognize the constructed nature of online personas and the impacts of self-presentation and social comparison, promoting authentic engagement. Encouraging questioning of content authenticity and idealized standards builds resilience, leading to healthier online interactions. These public health initiatives aim to support adolescents in developing a balanced relationship with digital technologies, enhancing their mental wellbeing in the digital age.

Moreover, based on our findings and other studies, it is necessary for parents, educators, and mental wellbeing professionals to be aware of the potential risks associated with a great focus on self-presentation. By fostering environments where adolescents feel comfortable expressing their authentic selves, it may be possible to mitigate some of the adverse mental wellbeing outcomes identified in this and related studies.

### Conclusion

This study presents evidence that focus on self-presentation is significantly associated with reduced mental wellbeing among younger adolescents, moderated by gender. Girls reported higher levels of self-presentation and were more negatively affected than boys, which could indicate that they are especially vulnerable to the pressures of maintaining an idealized online persona. These findings are in line with previous research that has highlighted the risks of social comparison and the potential benefits of authenticity in self-presentation.

The findings emphasize the importance of targeted interventions in mitigating the adverse influences of focus on self-presentation on social network sites. Educational programs that foster digital literacy and authentic self-expression may serve as protective factors, especially for girls. However, the cross-sectional design of the present study limits causal inferences, and the reliance on self-reported data introduces potential biases. Another limitation is the lack of direct measurement of authenticity in self-presentation, which will restrict the full understanding of its role in mental wellbeing. Longitudinal designs in future research are needed, and the inclusion of measures of authenticity will help to clarify the mechanisms underlying these associations. Public health strategies should focus on the development of healthier social media habits in support of adolescent mental health.

## Data Availability

The full dataset analysed during the current study are not publicly available, as they contain sensitive information, and the ethical approval of the study and GDPR preclude public access to these datasets. Requests to access the dataset should be directed to the HBSC Data Management Centre (dmc@hbsc.org). Older HBSC-datasets can be explored directly in the browser. Creating tables or downloading data requires user registration. Researchers, educators, journalists, and policymakers are granted free access, and the data can be analyzed and downloaded at www.hbscdata.uib.no.
