# Supplementary material for: Online Personas: Associations Between Focus on Self-Presentation and Social Comparison on Social Media and Mental Well-Being in Early Adolescence
Source: Int J Public Health. 2025 Apr 30;70:1608425. doi: 10.3389/ijph.2025.1608425 (PMC12074912; doi:10.3389/ijph.2025.1608425)
Supplement: Supplementary file 1 [file DataSheet1.docx]

- Supplementary material –

Online personas: Associations between focus on self-presentation and social comparison on social media and mental well-being in early adolescence

# APPENDIX SA

**Table A1.** Results from Mokken scale analysis, individual items. Self-Presentation and Upward Social Comparison Inclination Scale (SPAUSCIS). Data from Health Behavior in the School-Aged children Study (HBSC; Norway. 2022).

| **Item** |  | **_____** | **Item short name** | **Scalability** |
| --- | --- | --- | --- | --- |
| I spend a lot of time and energy on what I post on social media |  |  | Time and energy | 0.56 |
| It is important for me to get many likes and/or comments on what I post on social media |  |  | Likes important | 0.66 |
| It is important for me to have many followers on social media |  |  | Followers important | 0.66 |
| I delete what I post on social media if it does not get enough likes or comments |  |  | Deleting posts with few likes | 0.63 |
| I retouch photos of myself to look better before posting them on social media |  |  | Retouching | 0.55 |
| What others post (photos/status updates/stories) makes me feel less content with myself and my own life |  |  | Others’ posts affect feelings | 0.54 |
| The response I get for what I post (photos/status updates/stories) is impacts how I feel |  |  | Response affects feelings | 0.64 |
|  |  |  | **Total** | **0.60** |

**Comment**: Results from the Mokken scale analysis indicate that all the individual items are strongly scalable, and this amounts to a total-H of 0.60. The analysis also demonstrated excellent reliability, with a Molenaar-Sijtsma coefficient of 0.90, Cronbach’s *α* of 0.90, and Guttman’s *λ*-2 of 0.90. The Automated Item Selection Procedure (AISP) was conducted with a lower bound of 0.5 for item scalability coefficients. All 7 items formed a single scale. This indicates a very strong, unidimensional scale structure according to Mokken scale analysis criteria. Unidimensionality was also evident in exploratory factor analysis strongly indicating a 1 factor solution with a ratio between the first and second Eigenvalue of 9.6 (one factor 4.1 vs two factors 0.4). The mean factor loading for a one factor model was 0.74 (min 0.64/max 0.86).

# APPENDIX SB

**Table A2.** Associations between individual item of Self-Presentation and Upward Social Comparison Inclination Scale (SPAUSCIS) and mental well-being. Data from Health Behavior in the School-Aged children Study (HBSC; Norway. 2022).

|  | **Unadjusted** | | | **Fully adjusted** | | |
| --- | --- | --- | --- | --- | --- | --- |
| **Item** | **Coefficient (β)** | 95% CI^1^ | p-value | **Coefficient (β)** | 95% CI^1^ | p-value |
| Time and energy | -2.0 | -2.8, -1.1 | **<0.001** | -0.42 | -1.2, 0.40 | 0.3 |
| Likes important | -2.7 | -3.5, -1.9 | **<0.001** | -1.0 | -1.8, -0.23 | **0.012** |
| Followers important | -2.2 | -3.1, -1.4 | **<0.001** | -0.73 | -1.6, 0.09 | 0.082 |
| Deleting posts with few likes | -3.0 | -3.8, -2.1 | **<0.001** | -1.4 | -2.3, -0.60 | **<0.001** |
| Retouching | -3.3 | -4.2, -2.4 | **<0.001** | -1.8 | -2.6, -0.89 | **<0.001** |
| Others' posts affect feelings | -5.2 | -5.8, -4.5 | **<0.001** | -3.0 | -3.8, -2.3 | **<0.001** |
| Response affects feelings | -3.8 | -4.6, -3.0 | **<0.001** | -1.5 | -2.4, -0.68 | **<0.001** |
| ^1^CI = Confidence Interval  Fully adjusted: Age, gender, subjective socioeconomic status (S-SES) and problematic social media use | | | | | | |

**Comment**: All individual items of SPAUSCIS were significantly associated with lower mental well-being in unadjusted analysis (all p-values < 0.05). In the fully adjusted analysis, however, the association between two of the items and mental well-being were rendered non-significant (‘Time and energy’ and ‘Followers important’). This is likely to be partly due to the lower reliability of the estimates when the independent variable is a single-item measure, and forms only one part of the measurement scale for a unidimensional construct. When using only part of a scale, we are probably capturing less of the construct’s full scope. This can lead to decreased validity and reliability compared to using the full scale. But even in the fully adjusted model, all point estimates are still negative and uniformly points towards a negative association between aspects of focus on self-presentation and mental well-being among adolescents.
